# Reuterin disrupts *Clostridioides difficile* metabolism and pathogenicity through reactive oxygen species generation

**DOI:** 10.1080/19490976.2020.1795388

**Published:** 2020-08-17

**Authors:** Melinda A. Engevik, Heather A. Danhof, Ritu Shrestha, Alexandra L. Chang-Graham, Joseph M. Hyser, Anthony M. Haag, Mahmoud A. Mohammad, Robert A. Britton, James Versalovic, Joseph A. Sorg, Jennifer K. Spinler

**Affiliations:** a Department of Pathology & Immunology, Baylor College of Medicine, Houston, TX, USA; b Department of Molecular Virology and Microbiology, Baylor College of Medicine, Houston, TX, USA; c Department of Biology, Texas A&M University, College Station, TX, USA; d Texas Children’s Microbiome Center, Department of Pathology, Texas Children’s Hospital, Houston, TX, USA; e Department of Pediatrics, Children’s Nutrition Research Center, Baylor College of Medicine, Houston, TX, USA

**Keywords:** Reuterin, probiotics, *Lactobacillus reuteri*, *Clostridioides difficile*, enteroids, organoids, oxidative stress, reactive oxygen species, metabolism

## Abstract

Antibiotic resistance is one of the world’s greatest public health challenges and adjunct probiotic therapies are strategies that could lessen this burden. *Clostridioides difficile* infection (CDI) is a prime example where adjunct probiotic therapies could decrease disease incidence through prevention. Human-derived *Lactobacillus reuteri* is a probiotic that produces the antimicrobial compound reuterin known to prevent *C. difficile* colonization of antibiotic-treated fecal microbial communities. However, the mechanism of inhibition is unclear. We show that reuterin inhibits *C. difficile* outgrowth from spores and vegetative cell growth, however, no effect on *C. difficile* germination or sporulation was observed. Consistent with published studies, we found that exposure to reuterin stimulated reactive oxygen species (ROS) in *C. difficile*, resulting in a concentration-dependent reduction in cell viability that was rescued by the antioxidant glutathione. Sublethal concentrations of reuterin enhanced the susceptibility of vegetative *C. difficile* to vancomycin and metronidazole treatment and reduced toxin synthesis by *C. difficile*. We also demonstrate that reuterin is protective against *C. difficile* toxin-mediated cellular damage in the human intestinal enteroid model. Overall, our results indicate that ROS are essential mediators of reuterin activity and show that reuterin production by *L. reuteri* is compatible as a therapeutic in a clinically relevant model.

## Introduction

The overuse and misuse of antibiotics has led to the world’s current antibiotic resistance crisis; an era where bacterial pathogens are quickly becoming unresponsive to available antibiotics. The Centers for Disease Control (CDC) estimates there are >2.8 million illnesses and more than 35,000 deaths caused by antibiotic resistant organisms each year in the United States.^1^ Modern medicine relies on effective antibiotics not only for treating bacterial infections, but also for preventing them after routine or complex surgical, dental, and obstetric procedures. This reliance coupled with the ability of bacterial resistance to outpace new antibiotic development has fueled annual death toll predictions of 10 million people by the year 2050 from antibiotic resistant infections.^2^ Both the U.S. National Action Plan to Combat Antibiotic-Resistant Bacteria^3^ and the Global Action Plan on Antimicrobial Resistance outlined by the World Health Organization^4,5^ have emphasized the requirement for increased investment in novel compounds and approaches to combat resistance and associated diseases.

The leading hospital-acquired infection in the U.S. is caused by *Clostridioides difficile*, a spore-forming gram-positive bacterium that results in an additional ~224,000 hospital-acquired illnesses, 12,800 deaths, and 1 USD billion in healthcare costs annually.^1^ *C. difficile* infects the gastrointestinal (GI) tract, particularly the colon, and causes severe diarrhea and life-threatening complications that include pseudomembranous colitis and toxic megacolon. A key risk factor for *C. difficile* infection (CDI) is the disruption of a healthy gut microbiome by broad-spectrum antibiotics.^6–8^ Susceptibility to antibiotic-associated CDI is driven by decreased microbiome diversity resulting in increased amounts of primary bile acids that promote *C. difficile* spore germination and lower concentrations of secondary bile acids that inhibit *C. difficile* growth.^9,10^ Antibiotic use also hampers colonization resistance by decreasing the abundance of gut microbes that protect against *C. difficile* invasion.^11^ Despite the close association with prior antibiotic use, the first-line treatment for CDI is administration of the antibiotics vancomycin or fidaxomicin.^12^ Due to the continued antibiotic-mediated disruption of the gut microbiome during treatment, 35% of patients experience recurrent CDI after treatment ceases and approximately 40% of these patients go on to suffer multiple recurrent episodes.^13^ Consequently, researchers are exploring a wide range of therapies to combat CDI, including defined microbial therapy, toxin binding agents, immunotherapies, and probiotics.^14,15^

Natural antimicrobial production by human-derived commensal bacteria is an area rich with potential for developing novel approaches to prevent antibiotic-associated disease. Specific strains of human-derived *Lactobacillus reuteri* secrete reuterin, a secondary metabolite with antimicrobial activity against *C. difficile* and other enteric pathogens.^16,17^ Reuterin is an isomeric mixture of 3-hydroxypropionaldehyde (3-HPA)^18,19^ and does not interfere with the growth of commensal lactic acid bacteria.^16,20^ Using germ-free mice mono-associated with *L. reuteri*, Morita *et al*. demonstrated that reuterin is produced by *L. reuteri in vivo*.^21^ Moreover, reuterin has many promising therapeutic characteristics including water solubility, efficacy in a wide range of pH, and resistance to proteolytic and lipolytic enzymes.^18,22^ Reuterin has been shown to suppress *C. difficile* invasion of a complex microbial community without impacting the broader community dynamics^23^ and to promote gut microbiome diversity.^24^ The apparent narrow-spectrum activity of reuterin among a microbial community, its potential for boosting microbial diversity, and the intrinsic (nontransferable) resistance of *L. reuteri* to antibiotics used to treat CDI^23^ all support the use of *L. reuteri* as an adjunct therapy for CDI prevention and treatment. Yet the mechanism(s) by which reuterin inhibits *C. difficile* growth remains uncharacterized.

Previous work has demonstrated that reuterin reacts with thiols using Ellman’s reagent and induces gene expression associated with oxidative stress in *E. coli*.^25^ However, it is unclear whether oxidative stress is involved in the inhibition of *C. difficile*. Additionally, the effects of reuterin at the different stages of *C. difficile* growth (spore or vegetative) and the impact of reuterin on *C. difficile* metabolism and metabolites have not been characterized. Although reuterin production has been demonstrated *in vivo*, little data exists regarding the effects of reuterin on human epithelium, in the presence or absence of *C. difficile*. To address these questions, we have characterized the effects of reuterin on each stage of the *C. difficile* life cycle and assessed the impacts on *C. difficile* carbon metabolism and metabolite production. To model the protective effects of reuterin, we co-cultured *C. difficile* with human intestinal enteroid monolayers in the presence of reuterin and monitored cellular dynamics in real time. This work is among the first to characterize the mechanism of reuterin in the pathogen *C. difficile* and to delineate the effects of reuterin and glycerol on the host epithelium.

## Results

### Reuterin inhibits pathogenic but not dormant forms of *C. difficile*

**Figure 1.**
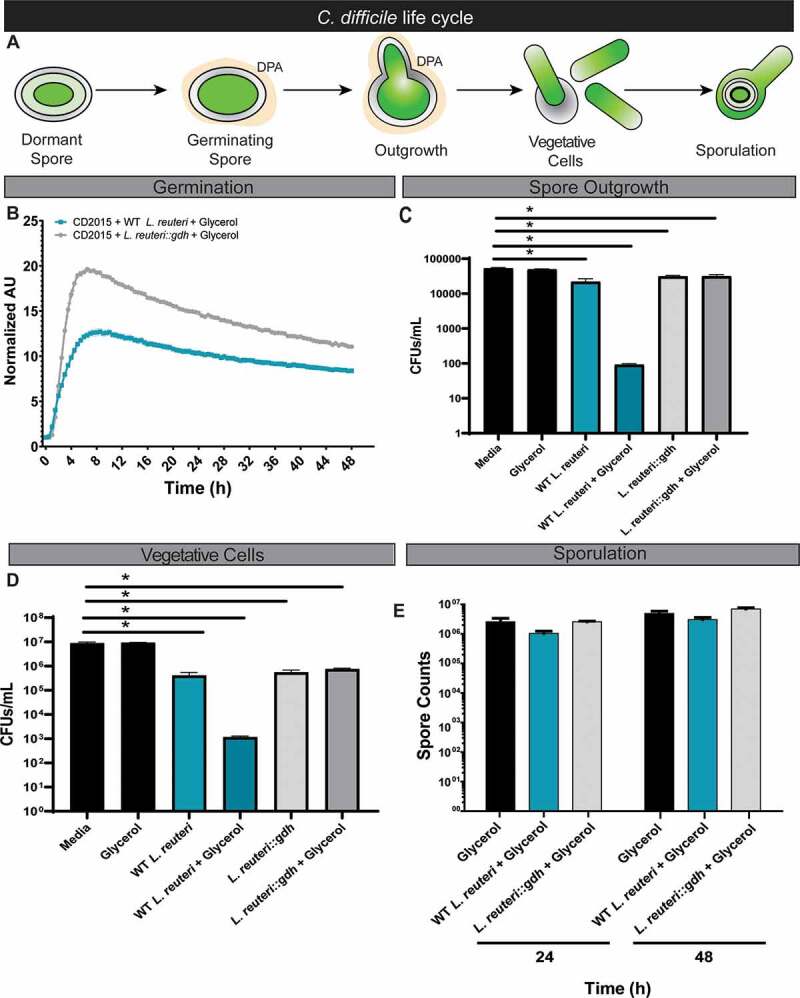
Reuterin production by *L. reuteri* affects vegetative *C. difficile* spore outgrowth, but not spore germination or sporulation. **A**. Diagram of the *C. difficile* life cycle. *C. difficile* enters the host as a dormant spore where interaction with primary bile acids induces spore germination and vegetative cell formation. Vegetative cells then colonize the host within the mucus layer of the intestinal epithelium. In adverse conditions, *C. difficile* will sporulate and repeat this life cycle. **B**. Germination of CD2015 was evaluated by measuring the release of DPA in the presence of primary bile acids, glycerol, and either wild-type *L. reuteri* 17938 or 17938::*gdh*. **C**. Spore outgrowth of CD2015 was evaluated by quantifying colony forming units (CFUs) in the presence of glycerol with or without 17938. **D**. The effects of reuterin on vegetative cells was tested with wild-type 17938 or 17938::*gdh* in the presence of glycerol. **E**. Sporulation of CD2015 in the presence of glycerol with wild-type 17938 or 17938::*gdh* was evaluated by quantification using phase-contrast microscopy.

Reuterin (also known as 3-HPA) is a secondary metabolite produced by human-derived *L. reuteri* during glycerol fermentation by the vitamin B_12_-dependent glycerol dehydratase (*gdh*).^26^ We previously demonstrated that active reuterin production by *L. reuteri* strain 17938 prevented *C. difficile* colonization of an antibiotic-treated human-derived fecal microbial community.^23^ To follow-up on our previous study^23^ and better understand the mechanism behind reuterin activity against *C. difficile*, we examined the effects of glycerol fermentation by wild-type *L. reuteri* 17938 and the isogenic *gdh* mutant incapable of producing reuterin, 17938::*gdh*, on the various phases of the *C. difficile* life cycle: (1) germination, (2) spore outgrowth, (3) vegetative cells, and (4) sporulation (Figure 1a).

The first step in initiating CDI is *C. difficile* spore germination (Figure 1a), a process that can be monitored by measuring the release of pyridine-2,6-dicarboxylic acid (DPA) from the spore core.^27,28^ We assessed whether *C. difficile* strain 2015 (CD2015) spores could germinate under conditions of active reuterin production by *L. reuteri*. Spores were incubated in the presence of wild-type *L. reuteri* 17938 or 17938::*gdh* in 10 mM glycerol, and DPA measured over 24 hr. The conversion ratio of glycerol to reuterin is approximately 1:1;^29^ 10 mM glycerol results in ~10 mM reuterin, which is greater than the 7.5 mM minimum inhibitory concentration found in *in vitro* monocultures (**Supplementary Figure 1**). Further, 10 mM reuterin has been previously shown to prevent *C. difficile* outgrowth in a microbial community.^23^ After 24 hr, we show that there was no significant reduction in DPA release from spores by wild-type *L. reuteri* under reuterin producing conditions as compared to the reuterin-deficient *gdh* mutant (Figure 1b). Additionally, when using ~10 mM purified reuterin to more directly assess reuterin activity, no significant changes in DPA release from spores were observed (**Supplementary Figure 2**). However, when examining *C. difficile* spore outgrowth, we found that outgrowth of vegetative cells from spores is significantly reduced to <99.1% after treatment with wild-type *L. reuteri* 17938 and glycerol at 24 hr (Figure 1c). Once *C. difficile* had transitioned to the pathogenic vegetative state, the addition of *L. reuteri* and glycerol significantly suppressed growth at 16 hr (Figure 1d), and this inhibition was dependent upon the ability of *L. reuteri* to ferment glycerol. Finally, we found that neither wild-type *L. reuteri* nor the *gdh* mutant either stimulated or inhibited *C. difficile* sporulation in the presence of glycerol (Figure 1e). Taken together, these data indicate that the reuterin-induced stress only impacts pathogenic vegetative phases (outgrowth and vegetative cells) of *C. difficile*. Additionally, reuterin does not affect either germination or formation of *C. difficile* spores. Importantly, reuterin activity does not induce the formation of *C. difficile* spores and dormant cell populations.

### Reuterin induces *C. difficile* oxidative stress

**Figure 2.**
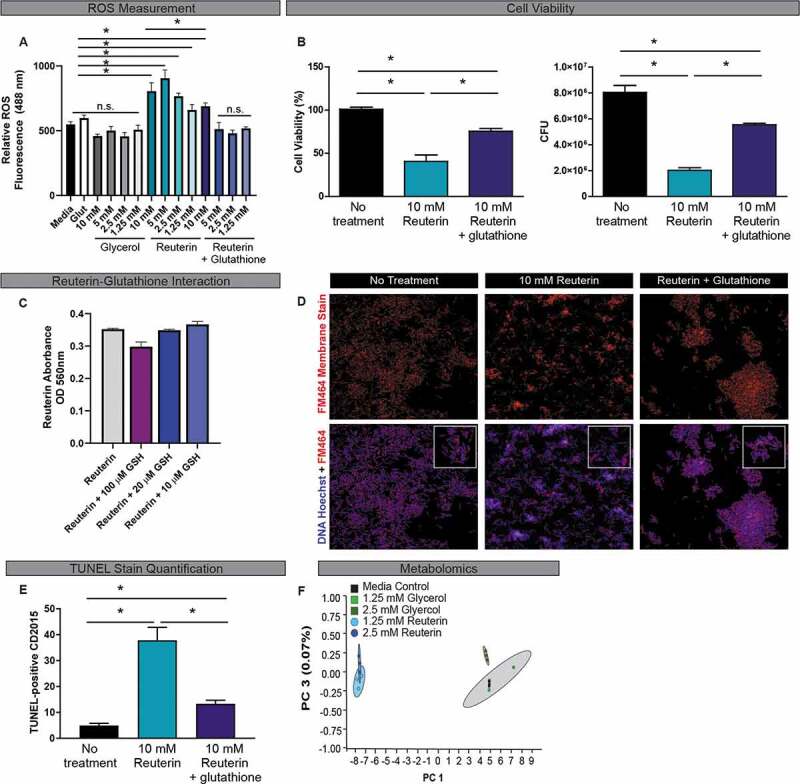
Reuterin induces reactive oxygen species (ROS) in **C. difficile**. **A**. ROS production by CD2015 after treatment with glycerol or reuterin with or without 20 µM glutathione was measured by dichlorodihydrofluorescein diacetate (DCFH-DA). **B**. Reuterin effects on cell viability was assessed by live/dead cell stain and confirmed by the quantification of CFUs. One-Way ANOVA, * p<0.05 (n=3 biological replicates, repeated 3 independent times). **C**. Reuterin-glutathione interactions were assessed by derivatization of tryptophan using a colorimetric assay. Data is represented by OD_560nm_ values (n=3 biological replicates). **D**. Effects of reuteirn-induced ROS on the cell membrane of CD2015 was measured by quantitation of cell membrane staining with or without 20 μM glutathione. Images were acquired at 100x on a Nikon Eclipse 90i. E. Effects of reuterin-induced ROS on DNA damage in CD2015 was evaluated by TUNEL staining with or without 20 μM glutathione and quantified using Image J (n=3 biological replicates). F. Mass spectrometry was used to produce metabolomics data from supernatants of CD2015 cultures treated with sublethal concentration of reuterin or glycerol and are represented as principle component analysis plots (n=3 biological replicates).

Previous studies in other bacterial species point to the role of reactive oxygen species (ROS) formation and overall oxidative stress in reuterin inhibition of bacterial growth.^25,30^ *C. difficile*, like many bacteria, possess detoxification enzymes like catalase, peroxidase, superoxide reductase, and superoxide dismutase to protect against ROS, indicating that oxidative stress is a key mediator of cell stress and death.^31^ Using a reuterin-null mutant above (17938::*gdh*), we have shown that the ability for *L. reuteri* to inhibit *C. difficile* growth relies on reuterin production through glycerol fermentation (Figure 1). To more directly assess whether reuterin induces ROS in *C. difficile*, we treated CD2015 for 30 min with various concentrations of purified reuterin and measured ROS by staining with dichlorodihydrofluorescein diacetate (DCFH-DA), a fluorogenic dye that measures hydroxyl, peroxyl, and other ROS activity within cells (Figure 2a). Compared to medium alone, glycerol (0–10 mM) had no effect on ROS generation in CD2015. However, reuterin treatment induced ROS activation, with 5 and 10 mM reuterin eliciting the greatest ROS activation (1.5- and 1.7-fold increase, respectively, compared to media control). Mammalian cells and some bacteria produce the powerful antioxidant glutathione to combat ROS. While *C. difficile* does not produce glutathione,^32^ *Clostridia* possesses glutathione transporters (gsiD)^32^ enabling glutathione uptake from the environment. Addition of 20 µM glutathione to 1.25, 2.5, 5, and 10 mM reuterin-treated CD2015 reduced the concentrations of ROS to that of medium control and glycerol treatment. A similar suppression of ROS was observed when CD2015 was pre-incubated with 20 µM glutathione, washed to remove extracellular glutathione, and subsequently treated with reuterin (data not shown), indicating that glutathione is internalized by *C. difficile*. Moreover, CD2015 cells treated with 10 mM reuterin and 20 µM glutathione had a significant increase in viability and CFU counts when compared to reuterin treatment alone (Figure 2b). To confirm these effects were not mediated by a direct interaction of reuterin and glutathione, we examined reuterin absorbance in the presence of varying concentrations of glutathione (Figure 2c). While a slight shift in absorbance was observed at high concentrations of glutathione (100 μM), reuterin absorbance was unchanged by lower glutathione concentrations (20 µM) used in our ROS experiments.

Because ROS can disrupt cell membranes and induce DNA damage,^33^ we next tested if reuterin-induced ROS altered these downstream targets in CD2015. *C. difficile* cells were treated with purified reuterin for 1 hr and cells immunostained with the cell membrane marker FM464. Immunostaining revealed loss of membrane continuity in 10 mM reuterin-treated CD2015 compared to no treatment controls. In contrast, addition of 20 µM glutathione significantly reduced alterations in the cell membrane at the 1 hr time point (Figure 2d). Reuterin-treated *C. difficile* was also evaluated for DNA damage in a TUNEL assay. A 1-hr treatment of CD2015 with 10 mM reuterin resulted in a 7.6-fold increase in TUNEL-positive cells compared to media control (Figure 2e); only a 2.6-fold increase in TUNEL-positive CD2015 cells was observed in the presence of 20 µM glutathione and reuterin (Figure 2e). These data demonstrate that reuterin-mediated oxidative stress in *C. difficile* results in cell membrane disruption, DNA damage, and cell death.

Reuterin-mediated ROS production in CD2015 was further supported by a global analysis of *C. difficile* metabolite production. The effect of reuterin on *C. difficile* metabolic profiles was characterized using global unbiased metabolomics analysis of CD2015 treated with sublethal concentrations of reuterin (1.25 and 2.5 mM) (Supplementary Figure 1). PCA plots of metabolomic data revealed a distinct shift in metabolites produced by CD2015 following incubation with 1.25 and 2.5 mM reuterin (figure 2f). These differences in metabolomic profiles were driven by statistically significant shifts in metabolites consistent with an oxidative stress response. Specifically, reuterin treatment resulted in increased relative concentrations of L-arginine, N-acetyl-L-methionine, butyl-4-hydroxybenzoate, and ethyl-4-hydroxybenzoate (**Supplementary Figure 3**), all of which have been linked to oxidative stress responses in bacteria.^34–37^ Equivalent concentrations of glycerol resulted in no metabolomic changes as indicated by co-clustering with samples from media controls.

### ROS activation by reuterin alters *C. difficile* metabolism

**Figure 3.**
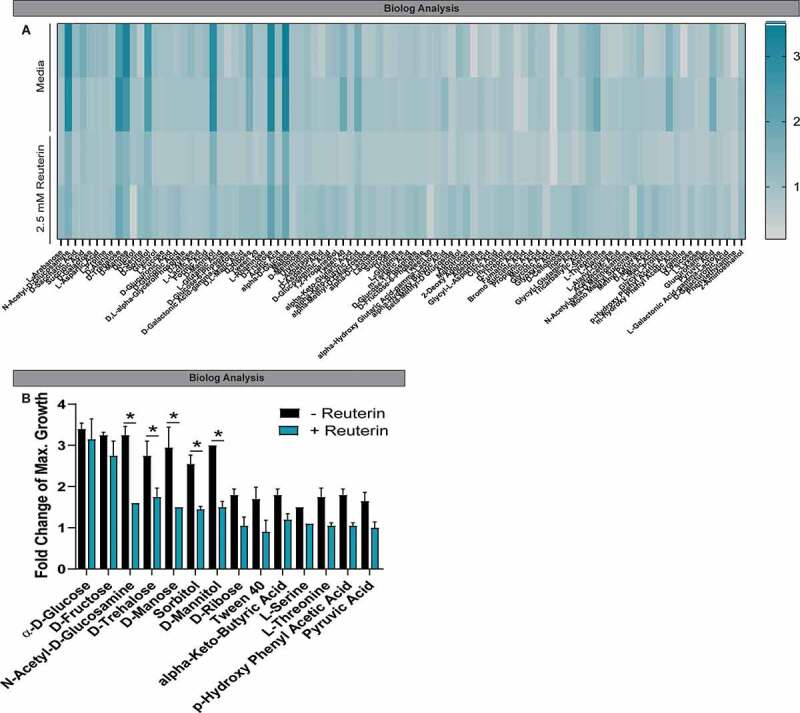
Reuterin influences the metabolic activity *C. difficile*. **A**. Growth of CD2015 cultures on various carbon sources in the presence of sublethal concentrations reuterin or glycerol were assessed using Biolog plates. Heatmap displays the maximal fold change of growth on the indicated compounds during the 16.5 hr growth curve in the presence and absence of 2.5 mM reuterin. **B**. Maximal growth curve peaks over the 16.5 hr time course were calculated and the fold change of growth over negative control is represented. Positive growth > 1.5-fold change; fold change = 1.0 indicates the same maximal value as the negative control (n=2 biological replicates).

Shifting primary carbon metabolism is one way bacteria counteract oxidative stress.^38–40^ Therefore, we assayed carbon utilization of CD2015 in the presence of 2.5 mM reuterin and screened 190 different carbon sources using Biolog Phenotype Microarrays (Figure 3, **Supplementary Figure 4**).^41^ We observed no general growth inhibition in medium containing glucose (**Supplementary Figure 4A**); however, five carbon sources displayed significant growth reduction in the presence of reuterin including host-associated sugars GluNAc and mannose, as well as dietary trehalose, sorbitol, and mannitol (Figure 3a-b). Additionally, trends in growth suppression were seen on other physiologically relevant carbon sources Tween, α-keto-butyric acid, L-serine, L-threonine, *p*-hydroxy phenyl acetic acid, and pyruvic acid (**Supplementary Figure 4B-D**).

**Figure 4.**
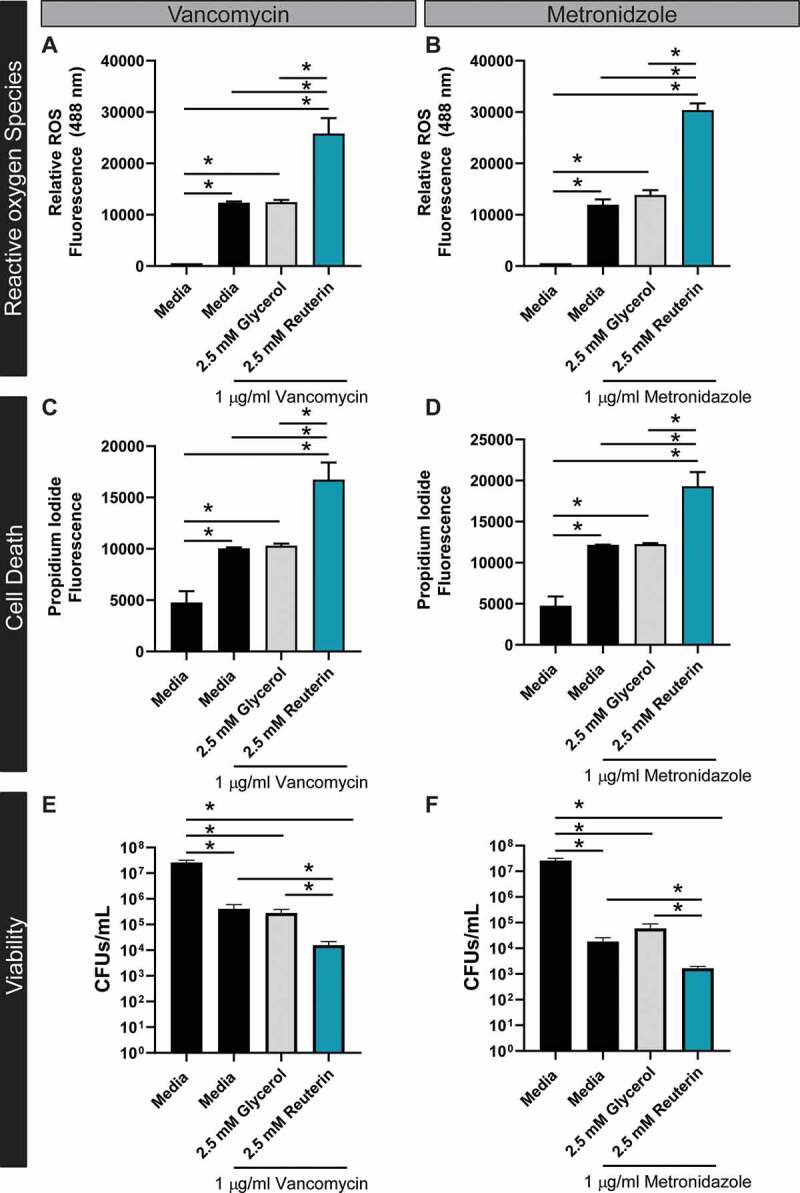
Reuterin works in synergy with antibiotics to promote ROS and cell death. Susceptibility of CD2015 to vancomycin (**A**) or metronidazole (**B**) was tested and ROS measured by staining DCFH-DA after treatment with glycerol or reuterin. Cell death was assessed by the dead cell stain Propidium iodide (excitation: 535, emission: 617) after exposure to vancomycin (**C**) and metronidazole (**D**). Cell viability was confirmed by CFUs counts for CD2015 treated with vancomycin (**E**) and metronidazole (**F**) (n = 3 replicates, repeated 2 independent times).

One mechanism of action common among bactericidal antibiotics involves ROS production.^42^ Reuterin-mediated oxidative stress and altered metabolism led us to reason that sublethal concentrations of reuterin may act synergistically with antibiotics. To address this question, vegetative CD2015 was incubated with or without 2.5 mM reuterin with 1 µg/ml vancomycin or metronidazole overnight. After exposure to antibiotics, CD2015 had elevated ROS staining; an effect that was enhanced by co-treatment of 2.5 mM reuterin (Figure 4a-b). Uptake of propidium iodide and CFUS were measured as a proxy for cell death (Figure 4c-f). After exposure to 2.5 mM of reuterin, *C. difficile* exhibited enhanced susceptibility to antibiotics used to treat CDI suggesting that even low concentrations of reuterin may improve the treatment of CDI by reducing metabolism of important physiological carbon sources and enhancing antibiotic sensitivity.

### Reuterin protects epithelial cells against *C. difficile* toxicity

Transcription of *C. difficile* toxin genes *tcdA* and *tcdB* is tightly linked to carbon metabolism. The catabolite control regulator CcpA controls expression of *tcdA* and *tcdB*,^43,44^ and we hypothesized that the shift in carbon metabolism in response to reuterin-mediated ROS may affect toxin production by *C. difficile*. Using ELISA, we measured both toxin A (TcdA) and toxin B (TcdB) production in the presence of either reuterin or glycerol over a 48-h period (Table 1). Both TcdA and TcdB production by CD2015 was reduced by 98.8% and 84.4%, respectively, in the presence of 10 mM reuterin. This is expected considering 10 mM reuterin inhibits CD2015 growth. However, we also observed that sublethal concentrations of reuterin decreased TcdB production (1.25 mM: 22.7%, 2.5 mM: 53.9%, 5 mM: 39.2%), but had less of an effect on TcdA production (1.25 mM: 0%, 2.5 mM: 2.8%, 5 mM: 8.4%). These data suggest that even very low concentrations of reuterin impact the production of TcdB, the primary virulence factor for *C. difficile*. We confirmed suppression of CD2015 toxicity using monkey kidney Vero cells, the gold standard for testing *C. difficile* toxins (**Supplementary Figure 5**). Vero cells respond to *C. difficile* toxins via toxin-induced rearrangement of the actin cytoskeleton that results in rounding of the fibroblast-like cells. Vero cells were incubated for 4 h with supernatants from CD2015 treated with reuterin or glycerol (1.25, 2.5, 5, or 10 mM). Consistent with the ELISA data, analysis of light microscopy images revealed no rounding present after incubation with supernatant from *C. difficile* cells treated with 10 mM reuterin, reduced cell rounding with supernatant from 5 mM reuterin treatment and no toxicity observed with supernatant from 2.5 or 1.25 mM reuterin-treated CD2015. No cell morphology changes were observed with any concentration of glycerol.

**Table 1.** *C. difficile* toxin analysis by ELISA.

Treatment	Toxin A	Toxin B	OD600 nm
Negative	0.06 ± 0.04	0.15 ± 0.00	NA
Media	4.00 ± 1.50E-3	0.92 ± 0.04	1.12 ± 0.24
10 mM Glycerol	4.00 ± 1.10E-3	0.85 ± 0.04	1.07 ± 0.33
10 mM Reuterin	0.05 ± 1.13E-3	0.14 ± 0.13	0.15 ± 0.02
5 mM Reuterin	3.66 ± 0.21	0.56 ± 0.09	0.44 ± 0.22
2.5 mM Reuterin	3.89 ± 0.14	0.43 ± 0.10	1.07 ± 0.26
1.25 mM Reuterin	4.00 ± 1.1E-3	0.71 ± 0.08	1.15 ± 0.21

**Figure 5.**
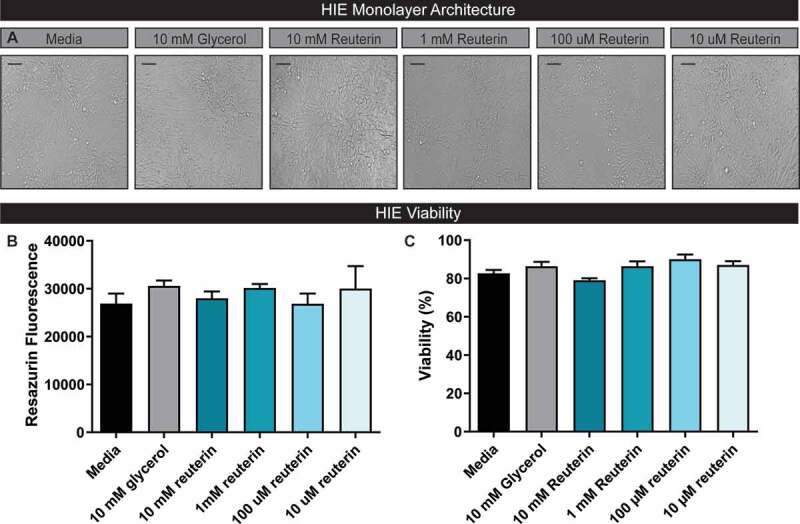
Reuterin does not negatively influence Human Intestinal Enteroid (HIE) architecture or cell viability. A.HIEs derived from the jejunum of healthy adults were grown as 2D monolayers and differentiated in CELLVIEW slides. Monolayer integrity and cell architecture was examined by light microscopy after 16 hr of incubation with glycerol or reuterin (scale bar =100 µm). B. To assess HIE cell viability, monolayers were incubated for with resazurin and fluorescence measured from the resulting supernatant. C. HIE cell viability was confirmed through trypan blue staining of dissociated monolayers (n=3, repeated 4 independent times).

Although immortalized Vero cells are commonly used to examine toxin activity, these cells do not recapitulate the morphology or function of the human intestine.^45,46^ In recent years, technological advances have enabled the propagation of primary intestinal epithelial stem cells in a system known as human intestinal enteroids (HIEs) or organoids.^47–49^ In this system, stem cells are propagated and differentiated into the various cell types found in the healthy human epithelium.^50–52^ HIEs represent a significant improvement over immortalized or cancer-derived cell lines in their ability to simulate intestinal physiology and are an ideal *in vitro* intestinal model to test reuterin efficacy. Since reuterin can induce ROS in bacterial cells, it was important to determine if reuterin would impart any negative effects on human intestinal cells. HIE monolayers were treated with 0–10 mM reuterin or 10 mM glycerol for 16 hr and viability was assessed by light microscopy, resazurin, and trypan blue staining (Figure 5a-c). By light microscopy, we observed the normal HIE architecture in the presence of reuterin and glycerol compared to medium controls (Figure 5a). Importantly, we observed that the monolayers were completely intact. Consistent with our microscopy findings, we found no change in viability by resazurin treatment of intact HIE monolayers (Figure 5b) or by trypan blue assessment of dissociated monolayers (Figure 5c).

**Figure 6.**
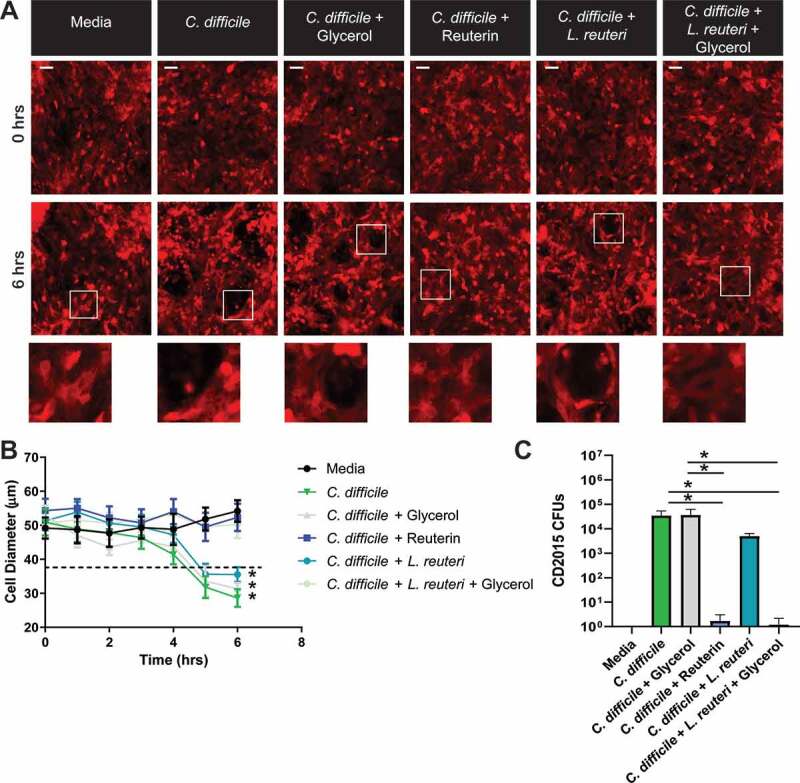
Reuterin limits HIE cell rounding in response to live ***C. difficile*** 2015. HIEs were transduced with the LifeAct-Ruby sensor to label F-actin (red). Cell rounding was visualized by live cell microscopy on a Nikon TiE with 20x Plan Apo (NA 0.75) differential interference contrast objective, using a SPECTRA X LED light source and ORCA-Flash 4.0 sCMOS camera (scale bar = 100 µm). **A.** Representative images of LifeAct-Ruby HIEs over time (0 and 6 hr) after exposure to live CD2015, CD2015 with 10 mM glycerol, CD2015 with 10 mM reuterin, CD2015 with live *L. reuteri*, or CD2015 with live *L. reuteri* and 10 mM glycerol. **B.** FIJI (Formerly Image J) software was used to calculate cell diameter over time (50 cells/image). **C.** Colony Forming Units (CFU) of CD2015 collected from HIE monolayers after overnight incubation. *P < .05, One Way ANOVA (n = 4 replicates per experiment, repeated 3 independent times).

*C. difficile* toxins are known to reduce the viability of HIEs through actin skeleton rearrangement.^53^ Analogous to the Vero cell rounding studies, we examined the effects of reuterin on *C. difficile* toxin-mediated HIE cell rounding. For robust visualization of the actin cytoskeleton, we used HIE cultures that stably express the F-actin fluorescent label LifeAct Ruby^54^ and monitored responses to CD2015 by live-cell fluorescence microscopy (Figure 6). No change in cell morphology was observed with media alone (control), while the addition of live *C. difficile* vegetative cells (10^7^ CFU) caused cell rounding after approximately 10 hr. Co-incubation of HIEs with both CD2015 and wild-type *L. reuteri* 17938 (10^7^ CFU) in the absence of 10 mM glycerol did not prevent *C. difficile*-induced cell rounding. However, when *L. reuteri* 17938 and 10 mM glycerol or 10 mM reuterin alone were added prior to the addition of CD2015, cell rounding was prevented up to 6 hr without visible cell damage (Figure 6a-b). Following a 16-h incubation, reuterin-induced *C. difficile* cell death (Figure 6c) as determined by CFU plating.

## Discussion

**Figure 7.**
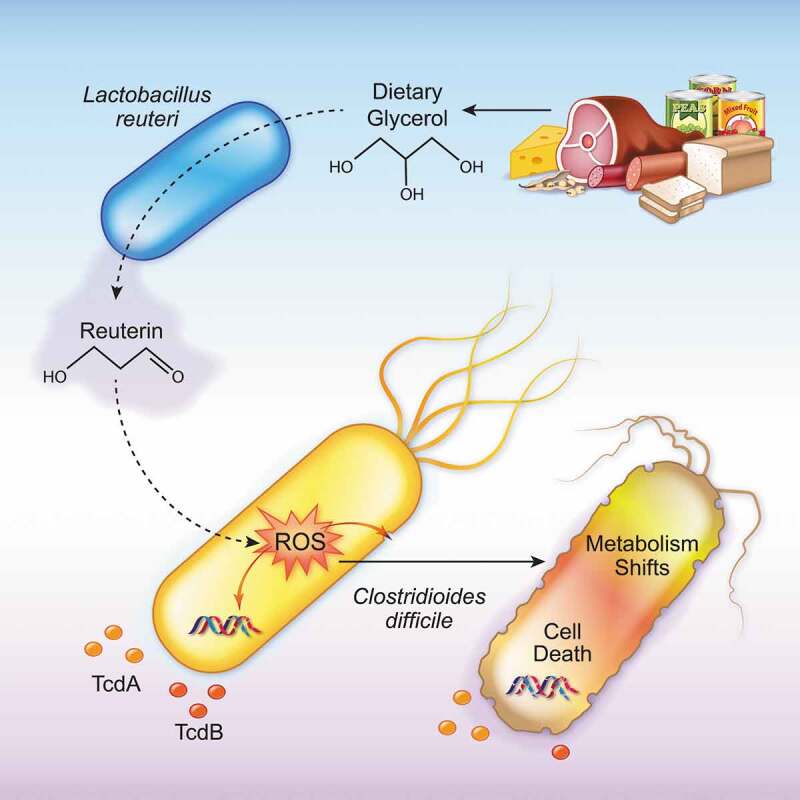
Model Diagram demonstrating that *L. reuteri* can convert dietary glycerol to reuterin, which activates reactive oxygen species (ROS) in *C. difficile*, causing shifts in metabolism, toxin production and cell death.

We have previously shown that active reuterin production by *L. reuteri* exhibited narrow-spectrum activity against *C. difficile* in a mixed microbial community and emphasized its promise as an adjunct therapy in CDI prevention.^23^ Here we characterized the effects of reuterin on the various cellular forms of *C. difficile* and shed light on reuterin’s mechanism of action against *C. difficile*. We predict that once inside the *C. difficile* cytoplasm the highly reactive aldehyde group of reuterin reacts with select compounds like flavoenzymes^55^ causing oxidation. The downstream effects of ROS production then disrupt the cell membrane and drive DNA damage^33^ resulting in cell death. Consistent with this hypothesis, we show that reuterin inhibits the growth of metabolically active forms of *C. difficile* by inducing the generation of ROS that results in a shift in carbon metabolism followed by a reduction in toxin production (Figure 7). Metabolically inactive forms of *C. difficile* are not affected by reuterin. Importantly, reuterin does not induce dormancy; a critical point considering spores are essential to *C. difficile* persistence.

The role of reactive oxygen species in stress responses and microbial metabolism has recently emerged as a major component in combating infectious disease.^38^ ROS compounds include hydrogen peroxide, superoxide, and hydroxyl radicals; all of which induce intracellular damage.^56,57^ In principle, ROS-mediated damage may also stimulate additional cycles of ROS accumulation, thereby generating a self-amplifying process that terminates in lethal stress.^58^ Multiple studies have recently indicated that ROS is a key pathway in the bactericidal activity of antimicrobial compounds, specifically antibiotics.^59–61^ Moreover, it has been speculated that stimulating ROS in pathogens could enhance the efficacy of a broad range of antimicrobials.^58^ Herein, we demonstrate that reuterin, a reactive aldehyde that oxidizes thiols and primary amines,^18,25^ induces ROS in *C. difficile*. While previous work showed that reuterin induced the expression of oxidative stress genes in *E. coli*,^25^ the effects of reuterin on *C. difficile* had not been demonstrated. We show here that 10 mM reuterin, a concentration efficient at limiting *C. difficile* growth in a microbial community,^23^ induces lethal ROS. However, at sublethal and biologically relevant reuterin concentrations,^62^ we hypothesize that *C. difficile* is able to detoxify ROS by catalase, peroxidase, superoxide reductase and superoxide dismutase enzymes; genes for which *C. difficile* has been shown to possess.^31^ Our data indicate that although *C. difficile* is capable of surviving sublethal concentrations of reuterin-induced ROS, this survival results in altered metabolism, reduced toxin production, and increased susceptibility to the commonly used antibiotics vancomycin and metronidazole.

We have shown that sublethal concentrations of reuterin alter the metabolism of *C. difficile* and this may influence the host’s ability to protect against CDI. A key step in colonization is the ability of a microbe to successfully acquire nutrients allowing for growth and establishment within the host. The intense competition for nutrients within the intestinal milieu has been suggested as a principal barrier to infection which combined with other host defenses prevents *C. difficile* from causing infection. However, when the microbiome is disrupted and nutrient competition reduced,*C. difficile *can establish a niche and cause infection. Nutrients key to *C. difficile* survival in the GI tract are glycan oligosaccharides including N-acetyl-glucosamine, amino acids that can be fermented via the Stickland reaction, and organic acids.^63^ Here we demonstrate that reuterin exposure, even at sublethal concentrations, impairs the ability of *C. difficile* to grow on these compounds. Recent studies suggest that the ability to utilize simple sugars and sugar alcohols has selected for the clinical emergence and persistence of virulent *C. difficile* strains,^64,65^ and we found that reuterin diminishes *C. difficile* growth on these compounds as well.

Pathogens have devised multiple methods for evading colonization resistance. Toxin production by *C. difficile* induces inflammation and diarrhea in the host, further contributing to the loss of microbial diversity in the gut in favor of *C. difficile* infection. While the pathogenesis of CDI is multifactorial, virulence of *C. difficile* relies on the production of exotoxins TcdA and TcdB with TcdB driving disease severity.^66^ Our data show that sublethal concentrations of reuterin reduce concentrations of both TcdA and TcdB produced by *C. difficile*; an effect we reason may be driven by ROS-induced shifts in carbon metabolism which may alter gene expression in *C. difficile*.^38,39^ Additionally, our work confirms that reuterin prevented toxin-induced cell rounding in human intestinal enteroids with no detrimental effects on the intestinal epithelium. Collectively these data reveal that reuterin can reduce *C. difficile* virulence by limiting toxin production without injuring the gut epithelium.

Although the exact pathways involved in *C. difficile*’s response to oxidative stress remain unclear, genome analysis has revealed several proteins putatively involved in the oxidative stress response,^67,68^ among which are rubrerythrins or rubrerythrin-like proteins. Rubrerythrins are non-heme di-iron proteins that serve as peroxide scavengers and are a particularly compelling candidate for potential adaptation to ROS. In *Clostridia*, rubrerythins are upregulated in response to hydrogen peroxide and oxygen,^69,70^ and overexpression has been demonstrated to enhance survival during exposure to these stresses^103^.

In conclusion, our data show mechanistically that reuterin generates ROS resulting in impaired metabolism of *C. difficile* that could decrease fitness in the GI tract and prevent *C. difficile* from effective nutrient competition. Reuterin decreased *C. difficile* virulence and protected HIEs by reducing toxin production. Furthermore, reuterin increased susceptibility of *C. difficile* to vancomycin and metronidazole, a synergistic effect that could potentially lower the dose and duration of antibiotics required to treat CDI. Taken together, these data are an indication of the promise reuterin holds through co-delivery of *L. reuteri* and glycerol as a probiotic adjuvant to traditional *C. difficile* therapies.

## Methods

### Bacterial strains and culture conditions

This study examined the inhibitory mechanism of reuterin production by *L. reuteri* DSM 17938 against *C. difficile* 2015. Routine anaerobic culturing of each strain was carried out at 37ºC in an anaerobic chamber (Anaerobe Systems, AS-580, Morgan Hill, CA) supplied with a mixture of 10% CO_2_, 5% H_2_, and 85% N_2_ for 16–18 h. *L. reuteri* and *C. difficile* were cultured in deMan, Rogosa, Sharpe (MRS; Difco, Franklin Lakes, NY) and Brain Heart Infusion media supplemented with 2% yeast extract, 0.2% cysteine (BHIS; BD Biosciences, Franklin Lakes, NJ), respectively. For work in a defined medium, *C. difficile* was cultured to stationary phase in BHIS and then subcultured in a Chemically Defined Minimal Medium (CDMM).^71,72^ All *C. difficile* cultures were normalized to a starting optical density (OD) at 600 nm of 0.1 for growth or 1.0 for inhibition studies using a Smartspec Plus spectrophotometer (Bio-rad Laboratories Inc). Colony-forming units (CFU) were counted from growth on BHIS agar plates. Chromosomal insertion in *L. reuteri* 17938::*gdh* was maintained by the addition of erythromycin (Erm; 10 µg/ml) to culture media. Specific culture conditions for individual experiments are detailed throughout.

### Reuterin production and quantitation

Production of reuterin by *L. reuteri* strains DSM 17938 and 17938::*gdh* was carried out as previously described.^16^ Briefly, cell pellets were collected at late log/early stationary phase, washed in sodium phosphate buffer, and resuspended in glycerol to ~1.5 x 10^10^ cells ml^−1^. Glycerol suspensions were incubated anaerobically at 37°C for 1 h, supernatants collected by centrifugation, filter sterilized, and stored at 4°C until further use. Reuterin stocks were quantitated indirectly through glycerol analysis measurements made by the Stable Isotope Core Laboratory of the Children’s Nutrition Research Center. Glycerol concentrations were determined for starting material before and after reuterin production using reverse isotope dilution methodology employing [^13^C_3_]glycerol as internal standard following conversion to the triacetate derivative and GCMS positive chemical ionization (PCI) as previously reported.^73^ Briefly, 50 µL of sample was spiked with an equal volume of the internal standard (10 mM) into 4 ml Teflon-lined screw cap vial, vortexed, and fully dried in servant drier. Then, 100 mM of acetic anhydride in pyridine (2:1) was added, vortexed, and heated 60°C for 30 min. Tubes were left to cool down and then dried under a gentle stream of nitrogen. Each sample was constituted in 500 µL of ethyl acetate and transferred to GCMS vial. Aliquots of 1–2 µL of samples or standards were injected into GC-MS (HP 5890/HP5970; Hewlett-Packard, Palo Alto, CA) and an HP-1701 column (30 m × 0.25 mm × 0.25 μm; Agilent Technologies, Wilmington, DE). GC conditions: 70°C for 1 min. Ramp @ 30°C/min to 180°C for 3 min Ramp @ 50°C/min to 280°C for 5 min. Injector: 250°C. Detector: 280°C. Helium flow 1 cc/min Retention time: ~5 min. Positive chemical ionization mode using methane gas was used and selected ion monitoring of m/z 159 and162 applied to monitor for natural and [^13^C_3_]glycerol, respectively.^73^ Assuming a 1:1 conversion ratio of glycerol:reuterin,^29^ the concentration of reuterin was determined by subtracting the remaining amount of glycerol from the GC-determined concentration of the starting material.

Reuterin-glutathione interactions were analyzed via derivatization of tryptophan in our standard colorimetric assay.^16^ Reuterin samples were incubated with or without glutathione (100, 20, and 10 μM) for 30 min at 37°C. After the incubation, reuterin was twofold serial diluted in water in a 96-well plate to a final volume of 40 μL per well. Reuterin (80 mM–0.65 mM) was used to generate a standard curve. To all wells, 30 μL of 10 mM tryptophan-HCl solution was added. Then, 120 μL concentrated (12 M, 32%) HCl was mixed in. Plates were covered and incubated for 20 minutes at 37°C, then OD_560_ measured using a Biotek Synergy H1 plate reader.

### *C. difficile* spore formation and purification

*C. difficile* spores were generated and purified as previously described.^28,74^ Briefly, *C. difficile* 2015 was cultured on BHIS agar medium for 4 days, cells scraped into 1 mL sterile ice-cold water, and incubated at 4°C overnight. Cells were then washed in water, purified by sucrose gradient centrifugation, and stored at 4°C in water until further use.

To study the effects of reuterin production on *C. difficile* sporulation, CD2015, *L. reuteri*, and *L. reuteri::gdh* were streaked from frozen stock onto BHIS-TA, MRS, and MRS-erm agar medium, respectively. After overnight growth, a colony of *C. difficile* CD2015 was inoculated into 70:30 broth supplemented with 10% glycerol, and a colony of each *L. reuteri* strain was inoculated into MRS broth. After overnight growth, each culture was back-diluted into 70:30 broth supplemented with 10% glycerol (3 mL total volume), together, to a final OD600 = 0.05 of each strain. As a negative control, *C. difficile* CD2015 alone was back diluted to an OD600 = 0.1. Spores were counted by phase-contrast microscopy at 24 and 48 hours.

### *C. difficile* spore germination and DPA release assay

Spore germination was initiated and monitored using terbium fluorescence, as done previously.^27,75,76^ *C. difficile* spores were incubated with or without 1) *L. reuteri* 17938 (~1.0 x 10^7^ cells ml^−1^) and 10 mM glycerol, 2) *L. reuteri* 17938::*gdh* (~1.0 x 10^7^ cells ml^−1^) and 10 mM glycerol, or 3) 10 mM reuterin for 24 h at 37°C. Reactions were suspended in germination solution (10 mM Tris (pH 7.5), 150 mM NaCl, 100 mM glycine, and 10 mM taurocholic acid), heat shocked at 65°C for 30 min and placed on ice. A 5-µL spore sample was added to each well of an opaque 96-well plate containing 125 µL of germination solution supplemented with 800 µM TbCl_3_.^27,77^ When released, DPA complexes with terbium (Tb^3+^) and fluoresces. This process was monitored using a Molecular Devices Spectramax M3 fluorescence plate reader (Molecular Devices, Sunnyvale, CA) with the following settings: 270 nm excitation; 545 nm emission; 420 nm cutoff.

### *C. difficile* spore outgrowth assay

*C. difficile* spores were incubated with or without (1) *L. reuteri* 17938 (~1.0 x 10^7^ cells ml^−1^) and 10 mM glycerol, (2) *L. reuteri* 17938::*gdh* (~1.0 x 10^7^ cells ml^−1^) and 10 mM glycerol, or (3) 10 mM glycerol for 24 h at 37°C. Colony-forming units (CFU) were counted from growth on BHIS agar plates containing taurocholate.

### Toxin ELISAs

Toxin production by *C. difficile* 2015 was measured in response to glycerol or reuterin. *C. difficile* 2015 was either cultured in CDMM (1) for 48 h with or without glycerol or reuterin (0–10 mM) or (2) treated with glycerol or reuterin (0–10 mM) after 24 h and subsequently cultured for an additional 24 h (for a total of 48 h). Supernatant from these two culture methods was collected, sterile filtered, and examined by toxin ELISA according to the manufacturers’ protocols (Eagle Biosciences: cat# DFA35-K01; DFB35-K01). Data were collected at 450 nm on a Synergy H1 Microplate Reader (Bio-Tek Instruments, Inc.).

### Analysis of *C. difficile* carbon metabolism

*C. difficile* CD2015 was cultured in BHI medium (Difco) supplemented with 0.5% (w/v) yeast extract (Fischer Scientific) overnight (~16 hours) in an anaerobic chamber (5% hydrogen, 90% nitrogen, 5% carbon dioxide). Overnight culture was diluted 1:10 in the fully defined minimal media CDMM^71^ with or without 2.5 mM reuterin as indicated. Cell suspension (100 µl) was added to each well of Biolog Phenotypic Microarray plates (PM1 and PM2) and the plate sealed with an optically clear film (Fisher Scientific). Growth assays were performed in duplicate in a plate reader (Tecan) under anaerobic conditions with an optical density at 620 nm read every 10 minutes for 16 h. Statistical analysis was performed by Two-way ANOVA (with Tukey’s correction for multiple comparisons where appropriate) in GraphPad Prism Software (v. 7.04).

### Global scan of *C. difficile* metabolites

The effect of reuterin on *C. difficile* metabolite production was examined by HPLC and mass spectrometry. *C. difficile* was cultured in CDMM with glycerol or reuterin (0–10 mM) for 16 hr. Supernatants were collected, filter sterilized, and dried to completeness with a SpeedVac. Samples were resuspended in 0.5x vol cold methanol and analyzed chromatographically using a Shimadzu (Kyoto, Japan) Nexera-XR HPLC system with an SIL-20ACxr autosampler, a CTO-20AC column oven and two LC-20ADxr binary pumps. The column consisted of a Phenomenex (Torrance, CA) 1 mm x 100 mm C18 (2) Luna column with a 4 mm × 2 mm C18(2) guard column. Elution gradients were generated from an aqueous mobile phase (A) of H_2_O:formic acid (99.9:0.1 v/v) and an organic mobile phase (B) of acetonitrile:formic acid (99.9:0.1 v/v). Samples (5 µL) were injected onto the column and eluted with a constant 80 µL/min mobile phase flow rate for a total chromatographic run time of 20 min per sample.

Samples were then analyzed on a Thermo-Fisher Orbitrap Fusion mass spectrometer in the positive ion mode under the following conditions: 1.6 kV spray volt-age, ion transfer tube = 350 deg C, 200–1000 m/z precursor ion scan range, 60% S-lens RF level, data type = profile, MIPS = on, 2–4 charge states, data dependent mode = top speed, precursor priority = most intense, MS level = 2, isolation mode = quadrupole, 1.6 m/z isolation window, activation type = CID; 35% CID collision energy, detector type = orbitrap, scan range mode = auto, 120,000 orbitrap resolution, AGC target = 5.0e4, 60 ms maximum injection time, microscans = 1, tandem MS data type = profile. Data were acquired with Thermo Xcalibur (v3.0.63).

Metabolite identification, quantification, statistical analysis was performed using Proteome Software (Portland, OR) Scaffold Elements. The spectra were searched against the Scripps Institute METLIN tandem MS library and National Institute of Standards and Technology (NIST) metabolite spectral databases. Candidate analyte identifications were generated by matching experimental data to spectral library data using exact mass with a mass tolerance of 20.0 ppm. If both the experimental and library data contained MS2 spectra, MS2 peaks were matched between experimental and library spectra using a fragment mass tolerance of 0.1 Da. To gauge confidence in candidate analyte identifications, an Analyte ID score was calculated by subtracting library entry matches from individual features, incorporating mass accuracy, isotopic distribution, and fragmentation pattern. Analyte identifications that were identified with more ion types received a higher score than identifications made with fewer ion types. Each technical replicate groups’ intensities were normalized to align the median intensities and the inner quartile widths with a bilinear mapping in log space. Identifications were accepted if they could be established with an Analyte ID Score of 0.7, based on peaks with log10 intensity levels of 0.0 or higher which are identified in 1 or more samples.

### Mammalian tissue culture and MUC2 purification

To purify MUC2, the dominant mucin secreted in the colon, human LS174 T cells (ATCC CL-188) were grown in DMEM medium (ATCC) containing 10% fetal bovine serum (FBS) (Invitrogen) at 37ºC, 5% CO_2_. LS174 T cells were treated with 10 µM of DAPT (Sigma-Aldrich) to promote goblet cell differentiation and incubated rocking for 5 days to drive mucus secretion.^78^ Mucin proteins from LS174 T supernatants were collected by ETOH precipitation with protease inhibitors as previously described.^79^ MUC2 was purified from crude mucin by guanidinium chloride and cesium chloride gradient ultracentrifugation on a Beckman Coulter Ultra-Centrifuge (30.2 Ti rotor) as previously described.^80^ MUC2-containing fractions were identified by slot blot immunostaining with a MUC2 antibody (cat# NBP1-31231, 1:100 dilution, Novus), LI-COR Odyssey Blotting reagents, Odyssey imaging system, and Image Studio software (LI-CORE Biosciences). MUC2 positive fractions were pooled, dialyzed, lyophilized, and resuspended in Hanks Salt Solution Buffer (HSSB). MUC2 concentration was quantified by BCA assay.

### Reactive oxygen species analysis

The production of reactive oxygen species (ROS) by *C. difficile* 2015 after treatment with reuterin was evaluated using a peroxynitrite indicator, 20–70-dichlorodihydrofluorescein diacetate (DCFH-DA) (Sigma-Aldrich). To examine ROS, overnight cultures of *C. difficile* 2015 in BHIS were washed thoroughly in PBS and adjusted to OD^600nm^ of 1 in sterile anaerobic PBS. PBS-cultures were incubated with glycerol (1.25, 2.5, 5.0, and 10 mM), reuterin (1.25, 2.5, 5.0, and 10 mM), or reuterin and 20 µM glutathione, which is known to suppress ROS. As a positive control, *C. difficile* was also incubated with 500 μM H_2_0_2_. All cultures were incubated for 30 min at 37ºC anaerobically. Following treatment, DCFH-DA was added at a final concentration of 10 µM and incubated for 30 min at 37ºC anaerobically. Non-stained *C. difficile* PBS cultures served as a negative control. The fluorescence emission of DCFH-DA was measured using a Biotek microtitre plate reader with an excitation wavelength of 485 nm. The background fluorescence of PBS and autofluorescence of the bacterial cells incubated without the probe were measured to calculate the net fluorescence emitted. Experiment was conducted in triplicate, three independent times.

### Immunostaining of C. difficile following reuterin exposure

To assess downstream targets of ROS, membrane disruption, and DNA damage, in *C. difficile* 2015, bacterial cells were washed thoroughly in PBS and adjusted to OD_600_ _nm_ of 1 in sterile anaerobic PBS and treated with 10 mM reuterin or 10 mM reuterin and 20 µM glutathione. As a positive control, *C. difficile* was also incubated with 500 μM H_2_0_2_. All cultures were incubated for 1 hr at 37ºC anaerobically. For membrane staining, *C. difficile* was incubated with 5 µg/ml FM 4–64FX (ThermoFisher #F34653) for 1 min on ice. Treated cells were then centrifuged at 5,000 x g for 5 min and fixed in 4% paraformaldehyde (PFA) at room temperature for 1 hr. Bacterial cells were counter-stained with Hoechst 33342 (Invitrogen) for 10 min at RT. Fixed bacteria were mounted on slides with Fluoromount Aqueous Mounting Medium (Sigma-Aldrich # F4680) and imaged on a Nikon Eclipse 90i. For DNA damage, *C. difficile* was incubated with 4% PFA at room temperature for 1 hr and dried on slides. Cells were permeabilized, treated with TUNEL staining reagents as previously described^81^, and imaged on a Nikon Eclipse 90i. TUNEL stained images were quantified using FIJI (formerly known as ImageJ, National Institutes of Health) software by tabulating the mean pixel intensity in five regions per slide (n = 2 slides/experiment; performed two independent times).

### Antibiotic treatment of *C. difficile*

To determine if reuterin affected the susceptibility of *C. difficile* 2015 to commonly used antibiotics, *C. difficile* 2015 was cultured in CDMM for 24 hr and adjusted to an OD_600_ _nm_ = 1 in CDMM. Cultures were subsequently cultured anaerobically at 37ºC, for an additional 24 hr with or without 2.5 mM reuterin or antibiotics (vancomycin, metrionidazole). Following the incubation, *C. difficile* cells were washed in PBS and incubated with either DCFH-DA to measure ROS or propidium iodidie to assess cell death. Additionally, samples were taken and plated in dilution of BHIS plates to generate CFU levels. Fluorescence readings were collected at 450 nm on a Synergy H1 Microplate Reader (Bio-Tek Instruments, Inc.).

### Vero cell rounding assay

*C. difficile* toxin activity was assessed by Vero cell rounding. Vero (ATCC CCL-81) cells were obtained from ATCC and grown in Dulbecco’s Modified Eagle Medium (ThermoFisher) supplemented with 10% fetal bovine serum (FBS) at 37°C, 5% CO_2_. Vero cells were routinely tested for *Mycoplasma* using the Mycoplasma Detection Kit (Lonza, cat# LT07-518). For cell rounding assays, Vero cells were grown to confluency on 96-well plates. Once confluent, the medium was changed to DMEM without FBS and *C. difficile* CDMM supernatant was used to treat the Vero cells (final concentration of 50% *C. difficile* supernatant in DMEM). Cells were imaged after 4 hr incubation on a Nikon TiE inverted widefield epifluorescence microscope (Nikon) using a SPECTRAX LED light source (Lumencor). Fluorescence and transmitted light images were recorded using an ORCA-Flash 4.0 sCMOS camera (Hamamatsu), and Nikon Elements Advanced Research v4.5 software was used for data acquisition. For analysis, FIJI (Formerly Image J; National Institutes of Health) was used to define cell shapes and cell diameter was recorded in three regions/well, n = 4 wells; repeated two independent times. Cell rounding was defined as a > 50% reduction in cell diameter.

### Human intestinal enteroid monolayer generation

Jejunum HIE cultures (termed J3) were obtained through the Texas Medical Center Digestive Diseases Center (TMC DDC) Gastrointestinal Experimental Model Systems (GEMS) Core. J3 HIEs stably expressing LifeAct-Ruby were described previously^54^ and HIEs were maintained in culture conditions as described previously.^48,82^ HIE monolayers were prepared from three-dimensional cultures and seeded into optical-bottom 10-well Cellview chamber slides coated with dilute collagen IV (Sigma) as described previously.^54,82^ To assess viability, monolayers were incubated with varying concentrations of reuterin for 16 h, then incubated with the dye resazurin (7-hydroxy-3 H-phenoxazin-3-one 10-oxide) (Sigma Aldrich) at a final concentration of 44 µM for 2 h at 37°C, 5% CO_2_. Cell viability was measured by reading the fluorescence resulting from resazurin reduction to resorufin using a microplate spectrofluorometer at an excitation wavelength of 570 nm and an emission wavelength of 600 nm. Next, cells were dissociated in PBS containing 3 mM EDTA and 10 mM Glucose for 5 min at 37ºC, 5% CO_2_. Viability was measured by Trypan Blue staining and analyzed on a Countess Automated Cell Counter (Invitrogen).

For analysis of enteroid cell rounding in response to *C. difficile* toxins, HIE monolayers were grown to confluency on CELLview chamber slides (GreinerBio), medium changed to optically clear FluoroBrite DMEM media (Invitrogen) supplemented with 15 mM HEPES (Invitrogen), 1X sodium pyruvate (Invitrogen), 1X GlutaMax (Invitrogen), and 1X non-essential amino acids (Invitrogen). Next, 10^7^ live *C. difficile* cells were added in FluoroBrite DMEM with or without 10^7^*L. reuteri*, glycerol, or reuterin. The slide was secured in an Okolabs stage-top incubation chamber with CO_2_ mixing and humidity control and imaged on a Nikon TiE inverted widefield epifluorescence microscope (Nikon) with a motorized X, Y, and Z stage for software-controlled multi-position imaging. Videos were recorded with widefield epifluorescence using a 20X Plan Apo (NA 0.75) phase contrast objective, a SPECTRA X LED light source (Lumencor) and an ORCA-Flash 4.0 sCMOS camera (Hamamatsu). Nikon Elements v4.5 software was used for image acquisition and FIJI (Formerly Image J) was used for analysis.

### Statistical analysis

GraphPad Prism software (version 8) (GraphPad Inc.) was used to generate all graphs. Statistical analyses were made with a one-way ANOVA with the Holm–Sidak *post-hoc* test. All the data are presented as mean ± standard deviation, with differences between the groups considered significant at *P* < .05 (*).

## Supplementary Material

Supplemental Material
