# Supplementary material for: Reuterin disrupts Clostridioides difficile metabolism and pathogenicity through reactive oxygen species generation
Source: Gut Microbes. 2020 Aug 17;12(1):1795388. doi: 10.1080/19490976.2020.1795388 (PMC7524292; doi:10.1080/19490976.2020.1795388)
Supplement: Supplemental Material [file KGMI_A_1795388_SM2726.zip › Supplementary information/Supplementary Figure Legends.docx]

**Supplementary Figure Legends**

**Supplemental Figure 1. Reuterin inhibits *C. difficile* vegetative growth**. CD2015 cells were adjusted to OD_600nm_=0.1 in CDMM and growth monitored anaerobically at 37˚C over 24 hr in a spectrophotometer. Serial dilutions of reuterin (10, 7.5, 5, 2.5, 1.25, 0.625, 0.31, 0.15 mM) were added.

**Supplementary Figure 2.** **Impacts of reuterin on CD2015 germination**. CD2105 germination, was examined by incubating CD2015 anaerobically for 48 hr at 37˚C in the presence of primary bile acids (30 mM Glycine and 10mM Taurocholate) with or without 10 mM reuterin. DPA release was measured overtime using plate reader (SpectaMax M3).

**Supplementary Figure 3. Reuterin-mediated metabolite production by *C. difficile* supports an oxidative stress response**. CD2015 cultures were adjusted to an OD_600nm_ = 0.1 in CDMM and incubated with sublethal concentration of glycerol or reuterin (2.5, 1.25 mM) for 24 hr. Metabolites released into the supernatant were then examined by mass spectrometry (n=3 biological replicates). Statistically significant metabolites were identified using Scaffold Elements Software.

**Supplementary Figure 4.** **Impacts of reuterin on CD2015 growth on indicated carbon sources**. CD2015 was suspended in defined minimal medium (CDMM) in the presence and absence of 2.5 mM reuterin and growth was monitored by optical density over time in a plate reader. Results from well C1 on Biolog plate PM1 containing D-Glucose-6-Phospate are shown. Calculated area under the curve for Glucose = 70.47 (± 0.59), Glucose + reuterin = 92.37 (± 0.42) and negative control = 30.37 (± 0.09). The negative control well supports a small amount of growth on the amino acids present in CDMM.

**Supplemental Figure 5. Reuterin inhibits *C. difficile* toxin production.** Vero cells were grown as 2D monolayers in CELLVIEW slides. Vero cell monolayers were incubated for 4 hr with supernatant from overnight cultures *C. difficile* 2015 grown in CDMM with glycerol or reuterin (0-10 mM). Cell rounding was visualized by microscopy on a Nikon TiE with 20x Plan Apo (NA 0.75) differential interference contrast objective, using a SPECTRA X LED light source and ORCA-Flash 4.0 sCMOS camera. Representative images of Vero cells following treatment (scale bar =100 µm) (n = 6 per experiment, repeated 3 independent experiments).
