# Supplementary figures and images for: Reuterin disrupts Clostridioides difficile metabolism and pathogenicity through reactive oxygen species generation
Source: Gut Microbes. 2020 Aug 17;12(1):1795388. doi: 10.1080/19490976.2020.1795388 (PMC7524292; doi:10.1080/19490976.2020.1795388)

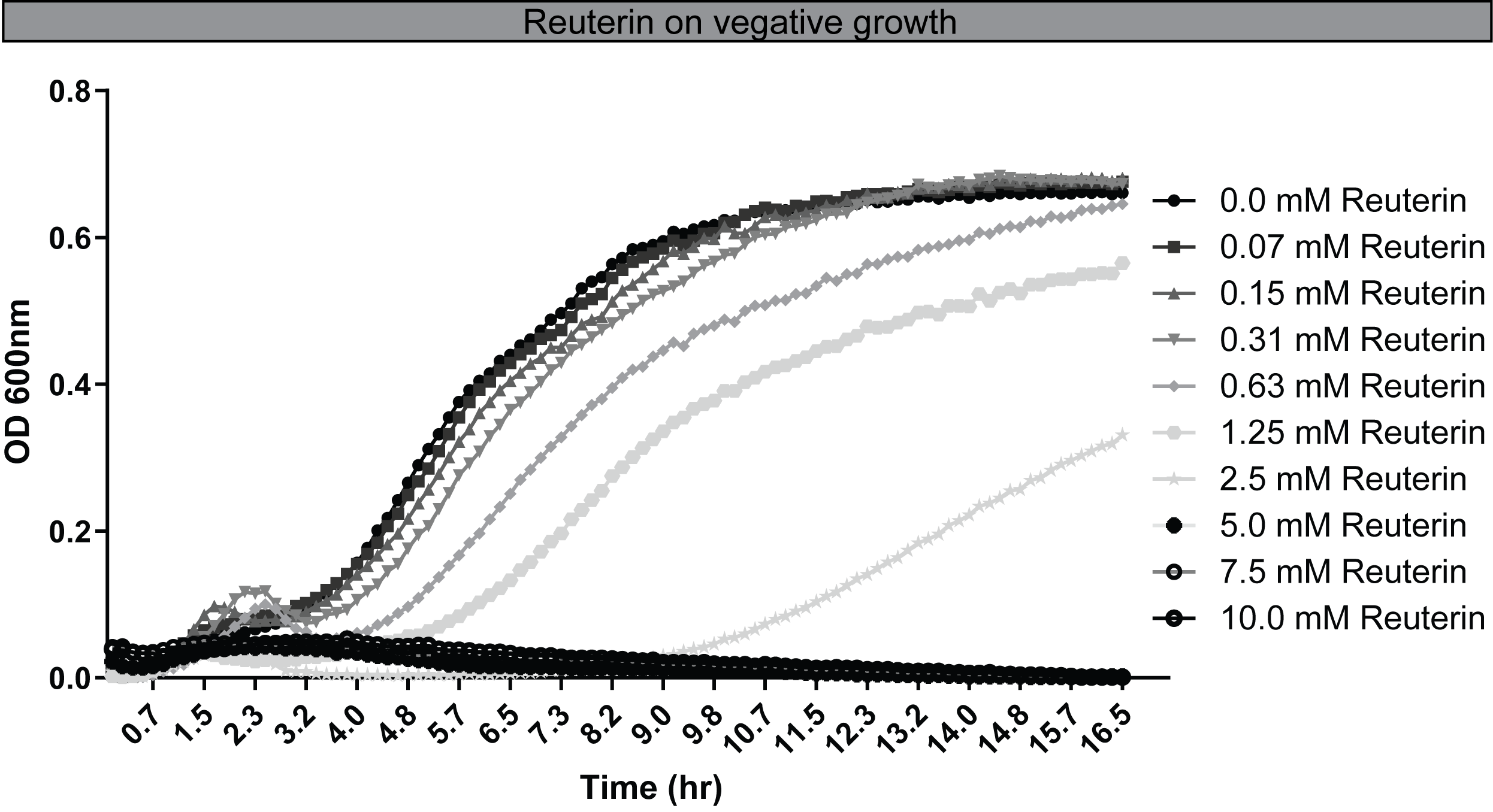

Supplement: Supplemental Material [file KGMI_A_1795388_SM2726.zip › Supplementary information/20Jun15 Supplemental Figure 1 Reuterin Growth black & white.tif]

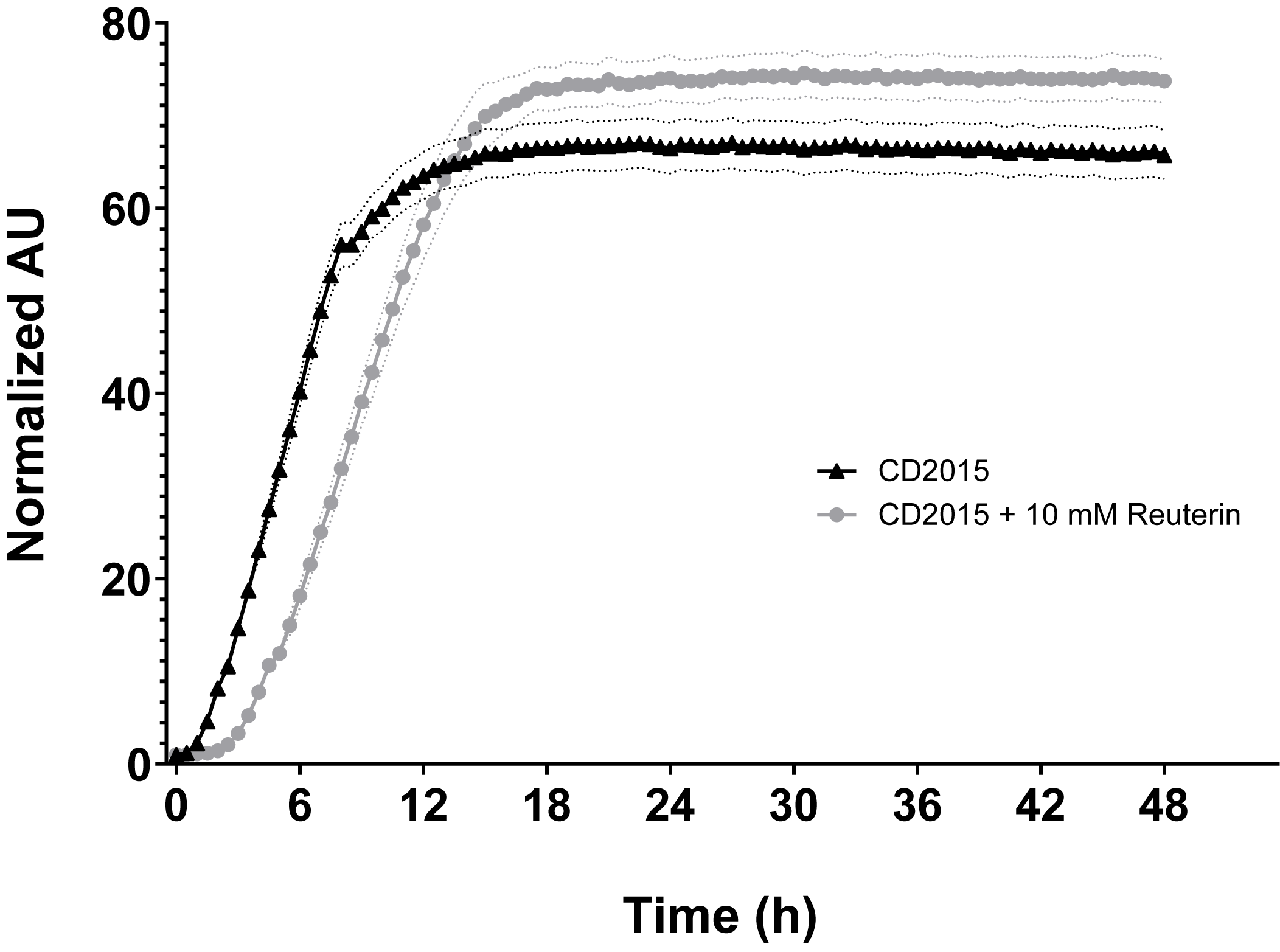

Supplement: Supplemental Material [file KGMI_A_1795388_SM2726.zip › Supplementary information/20Jun15 Supplemental Figure 2 Spore black & white.tif]

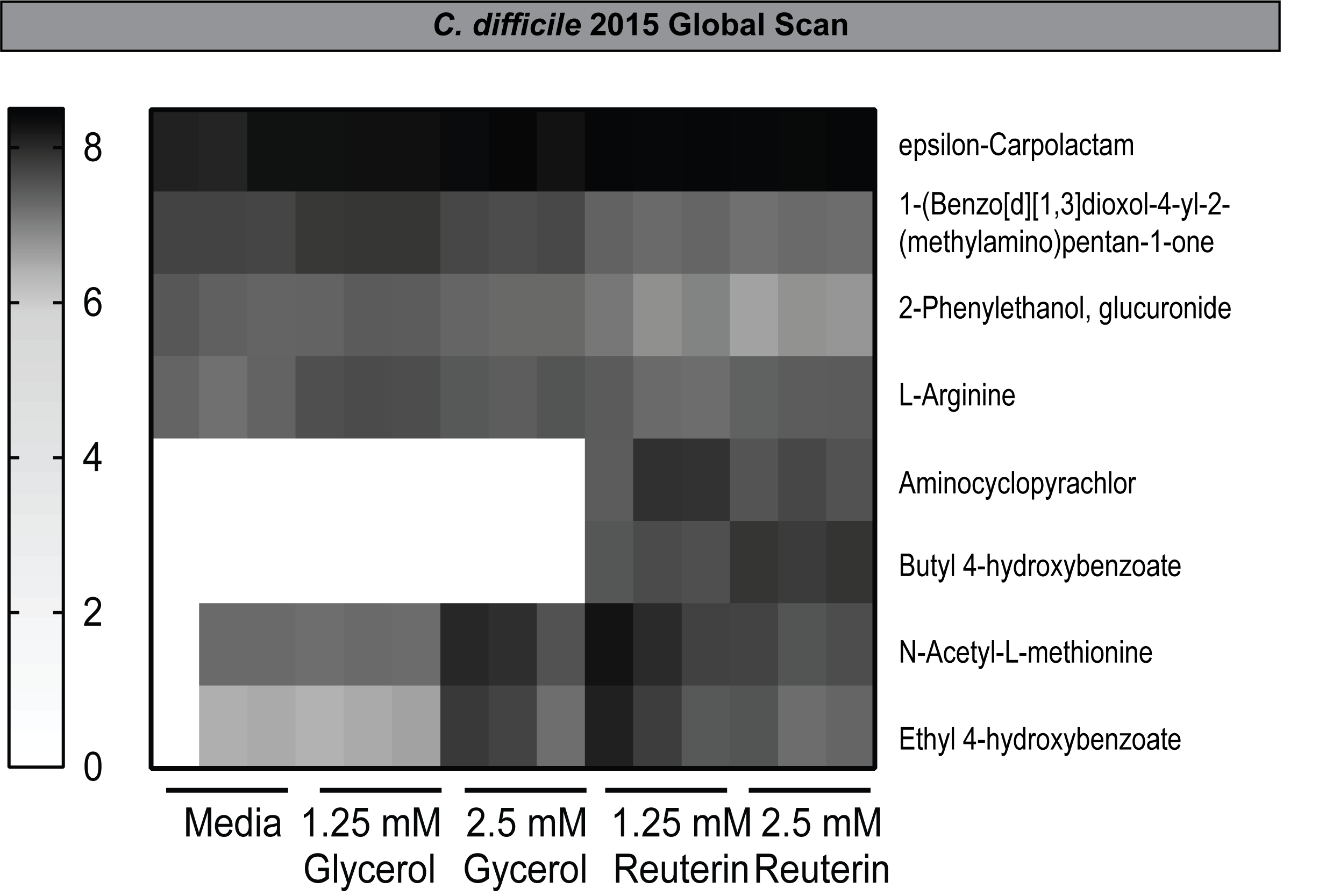

Supplement: Supplemental Material [file KGMI_A_1795388_SM2726.zip › Supplementary information/20Jun15 Supplemental Figure 3 Global Scan black & white.tif]

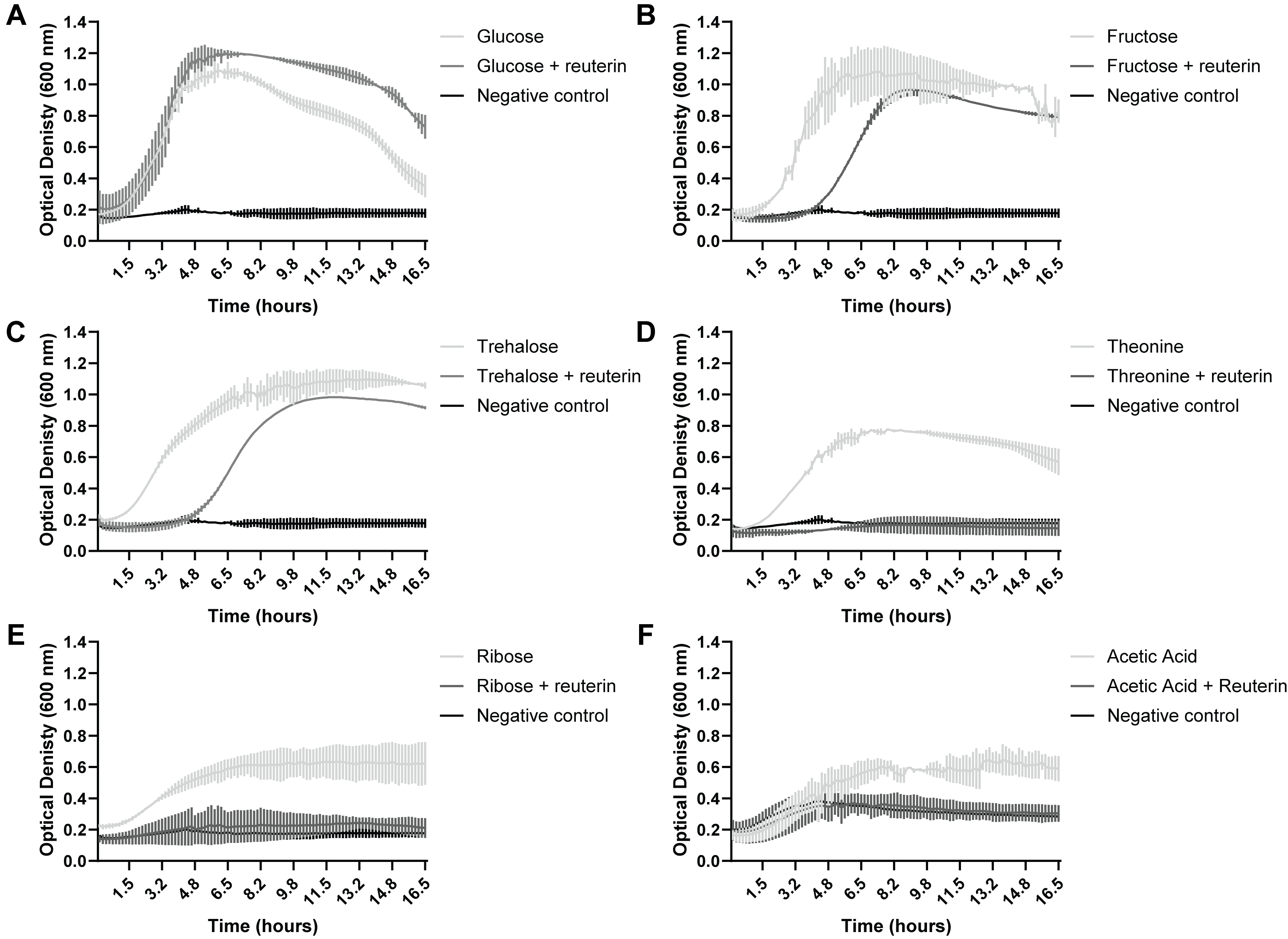

Supplement: Supplemental Material [file KGMI_A_1795388_SM2726.zip › Supplementary information/20Jun15 Supplemental Figure 4 Biolog black & white.tif]

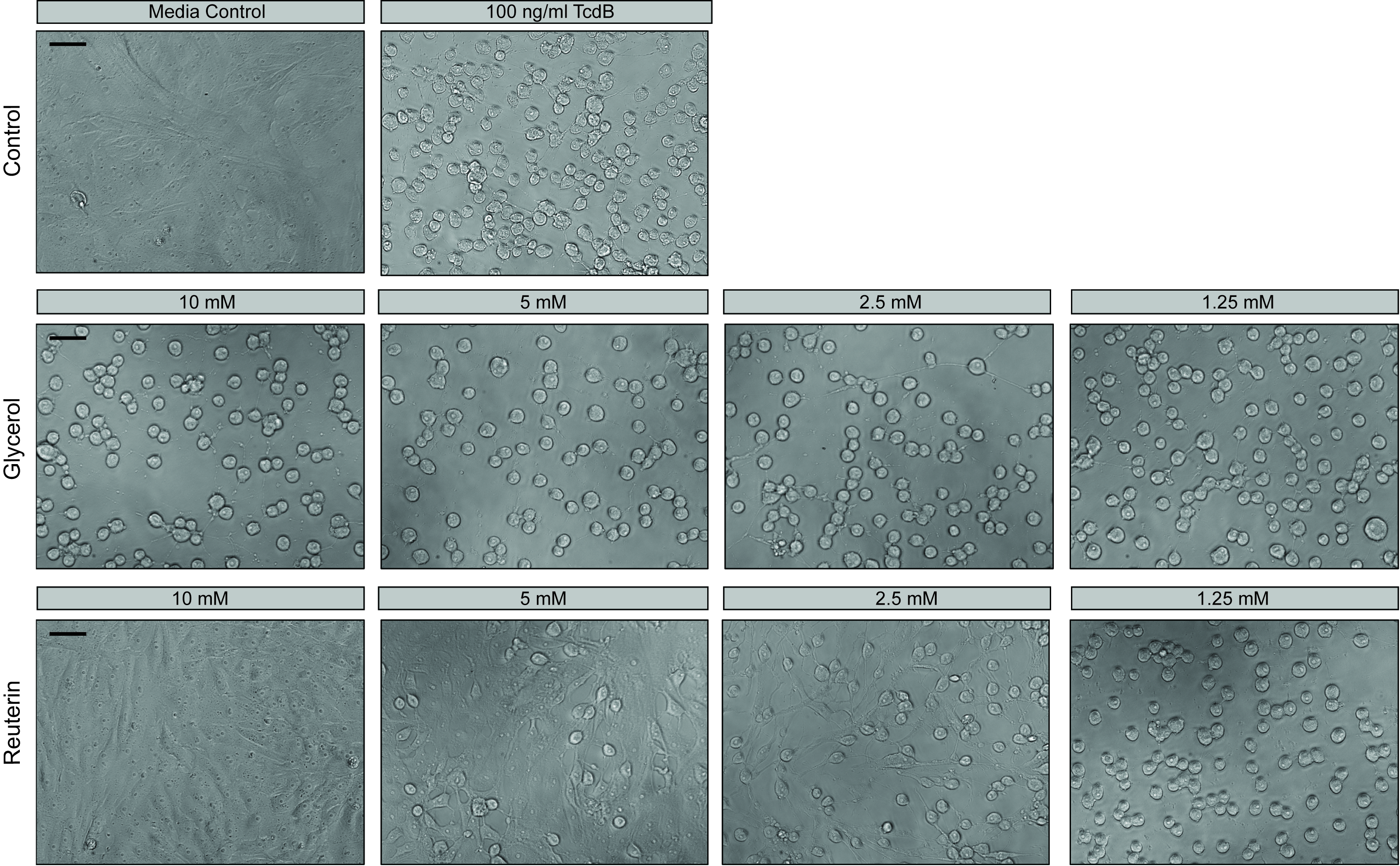

Supplement: Supplemental Material [file KGMI_A_1795388_SM2726.zip › Supplementary information/Supplemental Figure 5.tif]
